# Nephrotoxicity of perfluorooctane sulfonate (PFOS)—effect on transcription and epigenetic factors

**DOI:** 10.1093/eep/dvac010

**Published:** 2022-04-16

**Authors:** Yi Wen, Faizan Rashid, Zeeshan Fazal, Ratnakar Singh, Michael J Spinella, Joseph Irudayaraj

**Affiliations:** Biomedical Research Center, Mills Breast Cancer Institute, Carle Foundation Hospital, 509 W University Ave, Urbana, IL 61801, USA; Department of Comparative Biosciences, College of Veterinary Medicine, University of Illinois at Urbana-Champaign, 2001 S Lincoln Ave, Urbana, IL 61801, USA; Biomedical Research Center, Mills Breast Cancer Institute, Carle Foundation Hospital, 509 W University Ave, Urbana, IL 61801, USA; Department of Comparative Biosciences, College of Veterinary Medicine, University of Illinois at Urbana-Champaign, 2001 S Lincoln Ave, Urbana, IL 61801, USA; Department of Comparative Biosciences, College of Veterinary Medicine, University of Illinois at Urbana-Champaign, 2001 S Lincoln Ave, Urbana, IL 61801, USA; Department of Comparative Biosciences, College of Veterinary Medicine, University of Illinois at Urbana-Champaign, 2001 S Lincoln Ave, Urbana, IL 61801, USA; Cancer Center at Illinois; Carl R. Woese Institute for Genomic Biology, University of Illinois, 405 N Mathews Ave, Urbana, IL 61801, USA; Biomedical Research Center, Mills Breast Cancer Institute, Carle Foundation Hospital, 509 W University Ave, Urbana, IL 61801, USA; Department of Bioengineering, College of Engineering, University of Illinois at Urbana-Champaign, 1406 W Green St, Urbana, IL 61801, USA; Department of Comparative Biosciences, College of Veterinary Medicine, University of Illinois at Urbana-Champaign, 2001 S Lincoln Ave, Urbana, IL 61801, USA; Cancer Center at Illinois; Carl R. Woese Institute for Genomic Biology, University of Illinois, 405 N Mathews Ave, Urbana, IL 61801, USA

**Keywords:** PFOS, kidney, PPAR, epigenetic toxicity, histone methylation, transcription factors

## Abstract

Perfluorooctane sulfonate (PFOS) is a widespread persistent environmental pollutant implicated in nephrotoxicity with altered metabolism, carcinogenesis, and fibrosis potential. We studied the underlying epigenetic mechanism involving transcription factors of PFOS-induced kidney injury. A 14-day orally dosed mouse model was chosen to study acute influences *in vivo*. Messenger RNA expression analysis and gene set enrichment analysis were performed to elucidate the relationship between epigenetic regulators, transcription factors, kidney disease, and metabolism homeostasis. PFOS was found to accumulate in mouse kidney in a dose-dependent manner. Kidney injury markers *Acta2* and *Bcl2l1* increased in expression significantly. Transcription factors, including *Nef2l2, Hes1, Ppara*, and *Ppard,* were upregulated, while *Smarca2* and *Pparg* were downregulated. Furthermore, global DNA methylation levels decreased and the gene expression of histone demethylases *Kdm1a* and *Kdm4c* were upregulated. Our work implicates PFOS-induced gene expression alterations in epigenetics, transcription factors, and kidney biomarkers with potential implications for kidney fibrosis and kidney carcinogenesis. Future experiments can focus on epigenetic mechanisms to establish a panel of PFOS-induced biomarkers for nephrotoxicity evaluation.

## Introduction

Polyfluoroalkyl and Perfluoroalkyl substances (PFAS) are a group of synthetic compounds found as common environmental pollutants in various water sources, consumer products, and dust particles [1]. Perfluorooctane sulfonate (PFOS) is one of the most widespread PFAS compounds in the environment due to its extensive use as surfactants, refrigerants, soil and stain repellants, and in industrial and consumer products [2–4]. Studies in marine and wildlife revealed higher PFOS levels in aquatics and wildlife from more industrialized regions. Higher bioaccumulation in animals at the higher trophic levels of the food chain suggests the high bioaccumulation rate as a cause of the wide-spreading PFOS [5, 6]. The presence of fluorochemical residues in human blood, serum, or plasma from sampling populations indicates widespread exposure in humans [7, 8]. Compared to structurally comparable pollutants such as perfluorooctanesulfonamide (PFOSA), perfluorooctanoate (PFOA), or perfluorohexanesulfonate (PFHxS), PFOS was identified more frequently and at higher levels in human blood [8]. According to a review of biomonitoring studies, PFOS was identified in human sera at average levels of 10–75 ng/ml [9]. Because of their long half-life of 3.4–5.4 years in human sera, PFOS concentrations may stay elevated for longer periods [10, 11]. As a result, PFOS bioaccumulation in humans and animals could potentially have a significant impact on host metabolism, resulting in adverse health outcomes such as elevated levels of uric acid, cholesterol, lipids, glucose, blood pressure, hepatotoxicity, and renal toxicity [12–17].

The kidney is highly vulnerable to toxicants since it has the highest blood flow and the largest endothelial surface per organ tissue weight [18]. PFOS accumulates predominantly in the kidneys and liver through enterohepatic circulation and is excreted without biotransformation through the kidneys [9, 19]. Higher levels of lipids and glucose in serum can lead to their accumulation in kidney tissue that may cause chronic kidney disease (CKD) and diabetic renal injury, respectively [20]. Studies have demonstrated that PFOS exposure alters a variety of pathways associated with renal disease, which include peroxisome proliferator-activated receptors (PPARs), oxidative stress, and nuclear factor erythroid 2-related factor 2 (Nrf2) [21–23]. Analysis of data from the National Health and Nutrition Examination Survey suggested that elevated serum PFOS concentration was associated with a lower estimated glomerular filtration rate (eGFR) and an increased risk of chronic renal disease (CKD) among the US adult population [24]. Elevated PFOS levels can also cause kidney hypertrophy, microvascular pathology, and abnormal tissue proliferation in rats [25, 26]. PFOS was also found to cause diabetic kidney injury by altering purines and amino acid metabolism [27]. Changes in various genetic markers were observed, which led to inflammation and fibrosis in renal mesangial cells under diabetic conditions. Other studies have demonstrated the carcinogenic potential of PFOS, implicating its association with cancers of the liver [28] and bladder [29]. However, the carcinogenic potential of PFAS and its role in kidney cancer is still unclear. In this study, we will examine the molecular mechanisms through which PFOS imposes renal damage.

Epigenetic mechanisms play important roles in cells that are responding to environmental stimuli, including environmental toxicants. Despite past PFOS-induced nephrotoxicity studies, there is still a lack of knowledge on the role of epigenetic modifications in PFOS-induced kidney damage. Our previous studies have shown significant changes in the global DNA methylation landscape and in the gene expression profiles of epigenetic regulators after the exposure of PFOA and other endocrine-disrupting chemicals (EDC) [49, 50]. In this work, potential mechanisms by which PFOS contributes to renal damage and elucidation of key biomarkers of renal damage are highlighted. Specifically, we hypothesize that PFOS induces epigenetic alterations in the kidney, affecting the expression of transcription factors, including nuclear factors, consequently influencing the pathways of fibroblast activation, tumorigenesis, and metabolism of amino acids and lipids. The focus of our work is on epigenetic changes since they play an important role in the development of several renal disorders [30]. Past work shows that DNA methyltransferases (DNMTs) mediated hypermethylation of particular genes activates CKD and kidney fibrosis [31, 32]. Several investigations in damaged kidneys have also demonstrated that histone deacetylation suppresses transcription of BMP7 (a reno-protective protein) [33]. Likewise, abnormal histone methylation has also been associated with kidney damage [34]. Our present study will examine epigenetic mechanisms to shed light on cellular outcomes due to exposure to PFOS.

## Materials and Methods

### Chemicals and Test Reagents

PFOS was purchased from SynQuest labs (Alachua, FL, USA). Tween-20 was obtained from Sigma-Aldrich (St. Louis, MO, USA). The stock solution of PFOS was made in 0.5% Tween-20. Three doses of PFOS (5, 10, and 20 mg/kg/day) were prepared from the stock solution. A vehicle control for mice without PFOS was also prepared.

### Animals and Dosing Paradigm

The dosage levels were chosen based on prior research and reported levels of PFOS exposure in humans. It should be noted that the PFOS concentration in the body can reach up to 0.1 ug/g of body weight based on the weight of adults (60 kg), blood content (6400 ml), and serum concentration (1386 ng/ml) among employees in certain chemical industries [35]. Other research on various fish samples found that plaice liver and feral gibel carp had PFOS concentrations of 7.76 and 9.03 ug/g respectively [36, 37]. PFOS appears to be moderately hazardous in rats with a lethal dose of 250 ug/g body weight in some studies. Thus, to study the subacute effects of PFOS on the kidney, we chose three PFOS concentrations, 5, 10, and 20 mg/kg, for a 14-day period to consider both occupational and environmental exposure.

Adult CD-1 male mice were purchased from Charles River (Wilmington, MA, USA) and housed in ventilated polysulfone cages on 12 L:12D cycles at 25°C. Teklad Rodent Diet 8604 (Harlan) was fed to the mice and free access to clean water was provided. The University of Illinois Institutional Animal Care and Use Committee (IACUC protocol No. 19037) approved all animal protocols in accordance with the National Institute of Health guidelines. Triplicates of 60 days old CD-1 male mice were dosed with PFOS (5, 10, or 20 mg/kg/day) or vehicle control orally for 14 consecutive days. After 14 days of treatment, mice were euthanized, and kidney samples were immediately harvested and stored for further analysis.

### Liquid Chromatography–Mass Spectrometry

PFOS was extracted from kidney tissue following the methods of Mamsen *et al*. [38] with some modifications. Kidney tissue was mechanically homogenized in 70% acetonitrile with a 1**–**10 ratio for 2 min, and samples were incubated at room temperature overnight for maximum extraction. Samples were centrifuged at a maximum speed of 10 min, and the subsequent supernatant was carefully transferred into a glass vial. Meanwhile, standards of PFOS dissolved in 70% acetonitrile with concentrations of 0.625, 1.25, 2.5, 5, 10, and 20 µM were prepared to generate a standard curve. Extracted samples were analyzed using Ultra Performance Liquid Chromatography–Mass Spectrometry (UPLC–MS) SYNAPT G2-Si system (Waters Corp., Milford, MA, USA). The mobile phase was kept at 100% for 2 min, and 98% B for another 3 min. Column was conditioned with solvent A for the last 2 min. Electrospray ionization was set in negative ion mode for PFOS quantification by mass spectrometry.

### Hematoxylin and Eosin Staining

Hematoxylin and eosin (H&E) staining was performed on kidney samples to detect any alterations in pathological structure. Kidney samples from control and different dosages of PFOS-treated mice were immediately frozen with liquid nitrogen after collection. Kidneys were unfrozen and then incubated in 10% neutral buffered formalin (Sigma-Aldrich; St. Louis, MO, USA) for 24 hours at room temperature. After dehydration, kidneys were embedded in paraffin and cut into 5-μm thickness sections. Subsequently, H&E staining (Sigma-Aldrich, St. Louis, MO, USA) was performed on sectioned slides and imaged with a brightfield microscope.

### TUNEL Staining

Terminal deoxynucleotidyl transferase dUTP nick end labeling assay (TUNEL) was used to detect DNA breaks that occur during apoptosis. Therefore, this assay can be used as an indicator of cell apoptosis. Kidney samples were treated similarly as mentioned in the H&E staining section. Harvested kidneys were flash frozen and treated overnight in neutral buffered formalin. Then, kidney samples were fixed in paraffin and sliced into 10-μm sections. TUNEL assay (Abcam; Cambridge, MA, USA) was performed per instructions. Slides were counterstained with hematoxylin, and DNA breaks are detected as a brown stain under brightfield microscope.

### Gene Expression by Quantitative Real-Time Polymerase Chain Reaction

The total RNA from mouse kidney tissue samples was isolated with TRIzol™ Reagent (Thermo Fisher Scientific; Waltham, MA, USA). The concentration and quality of extracted RNA were tested with NanoDrop One (Thermo Fisher Scientific; Waltham, MA, USA). A complete complementary DNA (cDNA) library was synthesized with extracted RNA and via reverse transcription with the High Capacity cDNA Reverse Transcription Kit (Thermo Fisher; Waltham, MA, USA). PowerUp™ SYBR™ Green Master Mix (Thermo Fisher; Waltham, MA, USA) was used in combination with cDNA and DNA primers to perform quantitative real-time polymerase chain reaction (qRT-PCR). DNA primers were designed with NCBI RefSeq Transcripts and BLAST. qRT-PCR was performed on a Step One Plus Real-Times PCR System (Thermo Fisher, Waltham, MA, USA) with 96-well plates. Reactions were run in duplicate and normalized to messenger RNA (mRNA) expression of *Gapdh* on the same plate. Calculated ΔCT represents the relative mRNA expression of each target gene. Quantification of the expressed mRNA was calculated with 2–ΔΔCT method according to Schmittgen and Livak [39].

### Construction of Strand-Specific RNAseq Libraries

The DNA Sequencing Facility at the Roy J. Carver Biotechnology Center (University of Illinois Urbana-Champaign) performed the library construction and sequencing using the Illumina NovaSeq 6000. To assess RNA integrity, total RNAs were processed through a Fragment Analyzer (Agilent, CA, USA). The TruSeq Stranded mRNA Sample Prep kit (Illumina, CA, USA) was used to create RNAseq libraries. Enrichment of polyadenylated mRNAs was performed using Dynabeads® Oligo (dT) beads from 500 ng of high-grade DNA-free total RNA. This step was followed by chemical fragmentation of the mRNAs and an annealing step with a random hexamer and then converted to double-stranded cDNAs, which were then blunt-ended, 3ʹ-end A-tailed, and ligated to indexing adaptors. To avoid index switching, individual libraries were ligated to unique dual indexes. Using the Kapa HiFi polymerase (Roche, CA, USA), the adaptor-ligated cDNAs were amplified using 10 cycles of PCR. The resulting libraries were quantified using Qubit (ThermoFisher, MA, USA), and the mean library fragment length was calculated with a Fragment Analyzer. The libraries were further diluted to 10 nM and quantitated using qPCR using a Biorad CFX Connect Real-Time qPCR machine (Hercules, CA, USA) to ensure that barcoded libraries are correctly pooled, and maximum clusters are in the flow cell.

The barcoded RNAseq libraries that were pooled were placed onto a NovaSeq SP lane for clustering and sequencing. The sequencing of libraries was performed for 250 nt from either end of the fragments. The bcl2fastq v2.20 Conversion Software (Illumina, San Diego, CA, USA) was used to create and demultiplex the fastq read files.

### Bioinformatics Analysis of Rnaseq Data

The bcl2fastq v2.20 Conversion Software (Illumina, San Diego, CA, USA) was used to create and demultiplex the fastq read files. Initial quality control was done using FATSQC and low-quality reads were removed using Trimmomatic with sliding window 4:18, LEADING:28, TRAILING:28, and minimum length was kept to 35 nt. The resulting clean reads were then aligned to Mouse genome assembly NCBI GCF_000001635.27_GRCm39 using STAR aligner [40]. FeatureCount was used to count and assign the reads to genes [41]. Differentially expressed genes were identified using Limma R package with absolute fold change ≥2 and Benjamini–Hochberg False Discovery Rate (FDR) <0.05 was used to correct for multiple hypotheses [42]. Gene set enrichment analysis (GSEA) from the Broad Institute was performed to identify enriched gene sets using a maximum and minimum gene set size of 1000 and 15, respectively. The number of permutations was 1000 and the permutation type was set to gene set [43].

### Statistical Analysis

Statistical analysis was performed to distinguish any significant differences in gene expression between control and PFOS-treated mouse kidney samples. Data from the dose–response studies were analyzed with R statistics (R 4.1.0; R Core Team, 2021) and RStudio (1.4.1717, Rstudio Team, 2021). One-way analysis of variance (one-way ANOVA) was selected to assess differences across means of different dosage treatment groups. R function “aov” was performed for the one-way ANOVA. Further, to inspect the difference between each pair of means of treatment groups and control group, Duncan’s new multiple range test was performed post hoc with R function “duncan.test” after the installation of packages “agricolae.” One-way ANOVA significance levels were presented as marks above a line that grouped each gene expression in figures. Family-wise significance levels between control and treatment groups from Duncan’s post hoc multiple comparisons of means were presented as asterisk marks above corresponding bars in figures. Significance codes are respectively: “***” 0 < *P* ≤ 0.001, “**” 0.001 < *P* ≤ 0.01 “*” 0.01 < *P* ≤ 0.05.

## Results

### Assessment of Phenotypic Cytotoxicity of PFOS on Mouse Kidney

**Figure 1:**
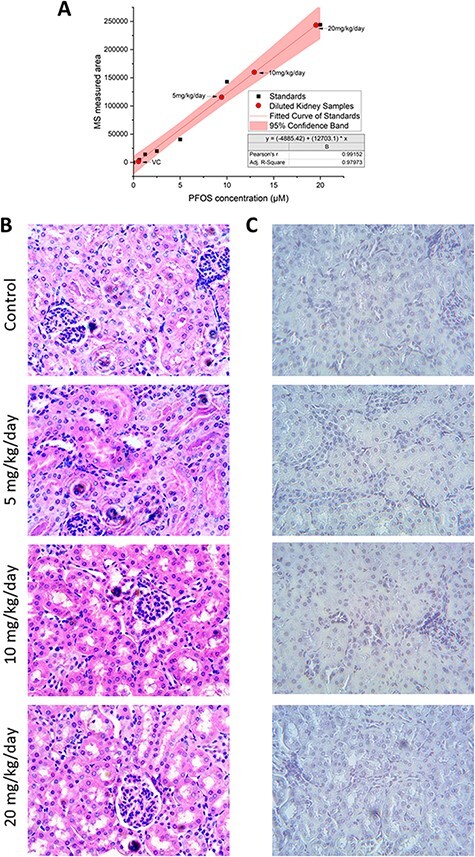
Kidneys of mice orally exposed with PFOS. (A) Presents the standard curve of PFOS constructed for UPLC–MS and the accumulated PFOS extracted from mice kidneys exposed to control and three levels of PFOS. (B) and (C) were imaged with 20× objective with scale bar of 50 μM. (B) H&E-stained mouse kidney sections. (C) TUNEL assay of mouse kidney sections

To assess the bioaccumulation levels of PFOS in mouse kidneys from mice that were orally dosed, PFOS was extracted from kidney tissues and analyzed through UPLC**–**MS. PFOS standards were used to generate a linear regression standard curve, shown as a solid line in Fig. 1A. The measured 10-fold diluted PFOS concentrations in the kidney tissue were plotted as red circular dots (Fig. 1A). The highest bioaccumulation noted in tissues was 197.78 μM at the dose of 20 mg/kg/day of PFOS.

H&E staining was performed to examine the pathological evidence of toxicity after PFOS exposure in mouse kidney tissue. Kidney samples from three independent mice from each treatment group were sectioned and imaged. One example from each group was presented in Fig. 1B. No significant difference was noted between control and treated kidneys. TUNEL assay was performed to assess any DNA breakage caused by apoptosis. One representative image from each treatment group was presented in Fig. 1C. No evidence was found to implicate the occurrence of DNA breaks during apoptosis in the tested samples.

### Effect of PFOS on Kidney Tissue and Pathway Expression

RNA sequencing was performed to investigate possible pathways dysregulated in the kidney upon PFOS accumulation. GSEA performed on control, 5, and 20 mg/kg/day PFOS-treated mouse kidneys revealed several significantly (FDR *q*-value <0.05) altered pathways due to PFOS exposure. Upon comparing low-dose PFOS (5 mg/kg/day) treated mice with the control, over 104 and 42 pathways were found to be significantly upregulated and downregulated, respectively. In high-dose PFOS (20 mg/kg/day) treated mice and controls, 77 and 17 pathways were found to be significantly upregulated and downregulated, respectively.

**Figure 2:**
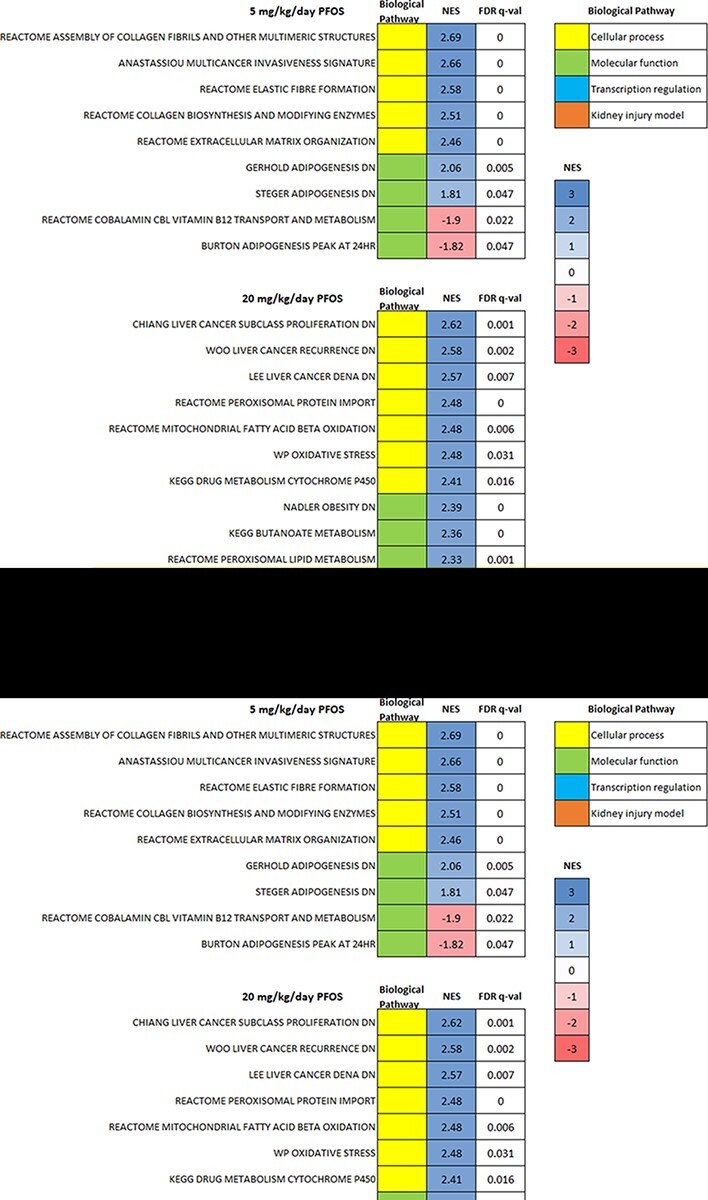
PFOS altered the expression of pathways for cellular process, molecular function, transcription regulation, and correlation with a kidney injury model. NES represents normalized enrichment scores; FDR represents false discovery rate

From the significantly altered pathways observed, we selected seven upregulated and two downregulated pathways from low-dose exposure group and 33 upregulated pathways from high-dose exposure group that are relevant to gene expression regulation in kidneys and in concordance with prior literature (Fig. 2). We also found a correlation between the kidneys of a mutated mouse model with renal defects and 20 mg/kg/day PFOS-treated group. All selected pathways’ absolute normalized enrichment scores were higher than 1.6. Selected pathways were grouped into four categories according to their functions: cellular process, molecular function, transcription regulation, and kidney injury model. For cellular process, strong evidence from GSEA in the 5-mg upregulated group supports PFOS-mediated alterations in fibrosis-related pathways, such as collagen biosynthesis and extracellular matrix organization. Multiple cancer-related pathways were also identified in the 20-mg PFOS-upregulated group, especially those related to liver cancer. Oxidative stress-related pathways were also activated after high-dose PFOS treatment. Most notably, several metabolism pathways were activated, especially for amino acids and lipids. Transcriptional regulation pathways altered predominantly included the PPAR pathways and tumor necrosis factor (TNF) pathways.

### PFOS Exposure Alters the Expression of Kidney Injury Markers and Transcriptional Factors Including Nuclear Receptors in Mouse Kidney

**Figure 3:**
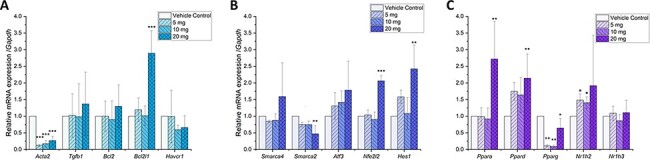
PFOS-induced gene expression changes in kidney injury markers and transcriptional regulators. Histograms show the relative mRNA expression of kidney injury markers (A), transcriptional regulation genes related to kidney damage (B), and nuclear receptors (C). The expression levels were normalized with *Gapdh*. *N* = 3 for all experimental data. PFOA treatment concentrations are shown as the mass (in the legend insert) per kilogram of mouse per day. Significance codes were ***0 < *P* ≤ 0.001, **0.001 < *P* ≤ 0.01, *0.01 < *P* ≤ 0.05, respectively

GSEA revealed a significant relationship between the effect of PFOS exposure on mouse kidneys and pathway alterations related to fibrosis and tumorigenesis. Next, we examined the genes contributing to fibrosis and tumorigenesis. The mRNA expression of five genes implicated as kidney injury biomarkers were examined through qRT-PCR analysis (Fig. 3A). Actine alpha 2 encoding *Acta2* mRNA expression decreased significantly (*P*-value < 0.001) in all treatment groups. The expressions of *Tgfb1* and *Havcr1*, which increased in acute kidney injury, did not change significantly [44, 45]. The mRNA expression of important cell death inhibitor *Bcl2l1* increased significantly at 20 mg/kg/day of PFOS treatment (*P*-value = 0.00071). While the mRNA expression of cell death repressor Apoptosis regulator Bcl-2 encoding *Bcl2* did not change significantly [46].

Transcription and repression of a majority of genes, including those found as kidney injury markers, are controlled by transcription factors. Therefore, we analyzed the expression of five transcription factors linked to kidney injury or carcinogenesis (Fig. 3B). The mRNA expression of probable global transcription activator SNF2L2 encoding *Smarca2* decreased after PFOS treatment at all concentrations and more significantly at the highest dosage (*P*-value = 0.0024). SMARCA2/BRM and BRG1/SMARCA4 are lost in approximately one-third of endometrial undifferentiated carcinomas [47]. In contrast, the expression of adenosine triphosphate (ATP)-dependent chromatin remodeler encoding *Smarca4* did not change significantly in our study. Our study showed a trend toward increased expression of *Atf3* after all treatment conditions. The expression of nuclear factor erythroid-derived 2-like 2 encoding *Nfe2l2* (*P*-value = 4.1e-5) and transcription factor HES1 encoding *Hes1* (*P*-value = 0.0059) both increased significantly after 20 mg/kg/day treatment.

As a family of ligand-regulated transcription factor, nuclear receptors play an important role in gene regulation of homeostasis and metabolism and are usually activated by steroids [48]. The mRNA expression of five nuclear receptors was examined, including PPAR (α, β/δ, γ), which had evidence of activation upon PFAS exposure from prior work [23]. Significant changes were observed after PFOS treatment (Fig. 3C). Expression of peroxisome proliferator-activated receptor alpha encoding *Ppara* significantly increased at 20 mg/kg/day treatment (*P*-value = 0.0079). The expression of peroxisome proliferator-activated receptor delta encoding *Ppard* also increased at all levels of treatment but increased significantly at the highest dosage (*P*-value = 0.0233). In contrast, the expression of peroxisome proliferator-activator gamma encoding *Pparg* decreased significantly at all treatment levels. Moreover, the expression of oxysterols receptor liver X receptor (LXR)-beta encoding *Nr1h2* increased with PFOS exposure.

### Epigenetic Toxicity of PFOS in Mouse Kidney

**Figure 4:**
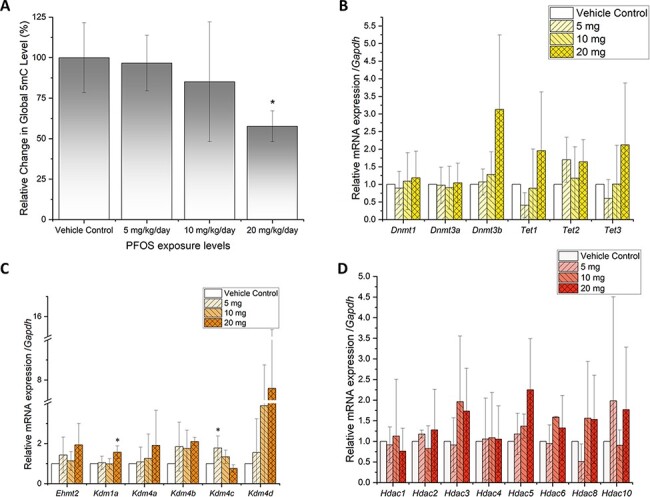
Influence of PFOS on the DNA methylation and gene expression of epigenetic regulators in mouse kidney. (A) Shows the relative global DNA methylation (5mC) levels in the kidneys exposed to PFOS relative to the vehicle control. Histograms show the relative mRNA expression of DNA methylation regulators (B), histone methylation regulators (C), and histone acetylation regulators (D). The expression levels were normalized with *Gapdh*. *N* = 3 for all experimental data. PFOA treatment concentrations are shown as the mass (in the legend insert) per kilogram of mouse per day. Significance codes were ***0 < *P* ≤ 0.001, **0.001 < *P* ≤ 0.01, *0.01 < *P* ≤ 0.05, respectively

To better understand the epigenetic effects of PFOS on genome-wide gene expression alterations, we examined the relative change in global 5mC levels and the gene expression of a number of DNA methylation, histone methylation and histone acetylation regulators. PFOS was found to decrease the global DNA methylation levels as shown in Fig. 4A. The highest dose exposure (20 mg/kg/day) caused significant (42%) hypomethylation in DNA (*P*-value = 0.0358). However, no significant changes were noted from the gene expression analysis of the three DNA methyltransferases, *Dnmt1, Dnmt3a,* and *Dnmt3b,* and three DNA demethylases, *Tet1, Tet2,* and *Tet3* (Fig. 4B).

**Figure 5:**
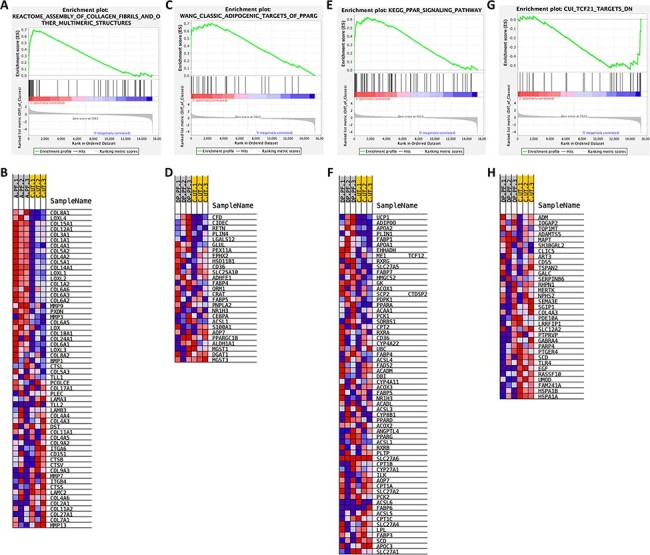
Selected pathways from GSEA of PFOS-exposed mouse kidney. Enrichment plot (A) and gene set heat map (B) for the assembly of collagen fibrils and other multimeric structures. Enrichment plot (C) and gene set heat map (D) for classic adipogenic targets of PPARG. Enrichment plot (E) and gene set heat map (F) for PPAR signaling pathway. Enrichment plot (G) and gene set heat map (H) for genes downregulated in the glomeruli of TCF21 KO mouse model

The gene expressions of histone methyltransferase *Ehmt2* and demethylases *Kdm1a, Kdm4a, Kdm4b, Kdm4c,* and *Kdm4d* were examined at all treatment levels (Fig. 4C). The expression of *Ehmt2* increased but not significantly at all treatment levels. *Kdm1a* expression significantly increased at 20 mg/kg/day treatment (*P*-value = 0.0351). *Kdm4c* expression significantly increased at 5 mg/kg/day treatment (*P*-value = 0.0321). No significant change was noted in the expression of *Kdm4a, Kdm4b*, and *Kdm4d*. None of the histone acetylation regulators, specifically histone deacetylases, examined had significant alterations in gene expression after PFOS treatment (Fig. 4D).

## Discussion

### Phenotypic Cytotoxicity of PFOS on Mouse Kidney

UPLC**–**MS quantification of PFOS in mouse kidneys demonstrated that PFOS accumulated proportional to oral dosing in the range of 5–20 mg/kg/day. In our experiments, the concentration of PFOS accumulated in the kidney tissue reached as high as 198 μM. Due to the slow elimination rate in mammals [51], such high concentration in kidney tissue can have a profound long-term impact on kidney function, especially on host metabolism and waste removal.

H&E staining and TUNEL assay did not detect significant differences between controls and PFOS-exposed kidneys. Since mice were harvested immediately after treatment, these results may indicate a less discernible cytotoxic impact of PFOS on kidney tissue in the short term. Future studies may be needed to examine its long-term effects. Nevertheless, PFOS exposure at these levels could still influence molecular pathways that potentially impact long-term kidney health.

### PFOS-Induced Alterations in Pathways and Markers of Kidney Injury

In contrast to the conventional manner of exploring potential candidate gene expression changes through single gene to gene comparison, GSEA is a robust approach to analyzing candidate pathway expression profiles. GSEA analysis of our acquired RNA sequencing data showed significant PFOS-mediated alterations in predefined gene sets, which can identify potential correlations to pathways [43]. Since most gene sets in GSEA were built within the human context, a homologous matching was performed to only include mouse genes with human homologs. This approach is possible and routinely employed due to the high genetic similarity between *Homo sapiens* and *Mus musculus.*

As shown in Fig. 2, numerous pathways were significantly altered after either 5 or 20 mg/kg/day treatment, including downstream metabolism, nuclear receptor mechanisms, and disease genesis pathways. We grouped the pathways into four main categories, cellular processes including disease genesis and peroxisomal processes, molecular functions including amino acid metabolism and lipid metabolism, transcription regulation including PPAR pathways and TNF pathways, and kidney injury model. For the 5 mg/kg/day PFOS-treated mice, four pathways related to fibrogenesis were upregulated significantly, including collagen biosynthesis, elastic fiber formation, and extracellular matrix organization (Fig. 2). PFOS-mediated fibrinogenesis is also supported by enrichment plots and the heat maps highlighting the altered expression of genes related to collagen biosynthesis (Fig. 5A and B). Lipid metabolism and vitamin B12 metabolism were also altered significantly after low-dosage PFOS treatment. According to Gai *et al*. [20], lipid accumulation can cause chronic kidney diseases. In the 20 mg/kg/day FPOS-treated mouse kidney, all three main categories of pathways were positively activated (Fig. 2). Within cellular processes, cancer proliferation, peroxisomal protein import, and oxidative stress pathways were upregulated. Studies have shown that PFOS induces oxidative stress in zebrafish embryos through Nrf2 and MAPK pathways [22] and through disrupting antioxidant equilibrium [52]. For molecular functions, adipogenesis, peroxisomal lipid metabolism, glycine metabolism, and multiple amino acids metabolism pathways were upregulated. PFOS and other PFAS such as PFOA-induced lipid metabolism disturbance have been observed in many *in vitro* and *in vivo* studies, both in human and in animals, but most often in the liver [49, 53, 54]. Nuclear lipid hyperaccumulation was also conjectured to play an important role in PFAS-induced toxicity [55]. Our study found amino acid metabolism pathway alterations, which corresponds to results found in a human metabolomic profiling study [53]. PPAR and TNF pathways were also strongly upregulated among other transcription regulation mechanisms. PPARs are a group of important transcription factors regulating cellular fate and metabolism [56] and could also contribute to tumorigenesis [57]. GSEA enrichment plots and heat maps for the PPAR pathway are presented in Fig. 5E and F. Moreover, increased histone methylation H3K4me3 of *TNF* has been found to be related to kidney inflammation [58]. Furthermore, we compared the gene expression profiles of the existing mouse kidney injury models and our PFOS-treated groups and found downregulated genes correlation between kidney glomeruli isolated from *TCF21* knockout (KO) mice and kidneys from 20 mg/kg/day PFOS-exposed mice (Fig. 5G and H). The *TCF21* KO mice die in the perinatal period with multiple renal defects [79]. This evidence supports the concordance between kidney defects and PFOS-induced kidney injury.

Motivated by the GSEA-indicated pathways, the mRNA expression of five kidney injury markers were studied via qRT-PCR (Fig. 3A). It is known that the expression of ACTA2 correlates with the inhibition of fatty acid oxidation [59]. Therefore, a significant downregulation of *Acta2* could be correlated to the activation of fatty acid oxidation. According to Kang *et al*., PPARs and PPAR gamma coactivator-1 are the key transcription factors that regulate fatty acid metabolism [59]. TGFB1 was found to induce clear profibrotic phenotypes characterized by the loss of mesenchymal markers such as ACTA2. Sustained expression of TGFB1 was found to be related to progressive kidney fibrosis [45]. However, we did not find any significant change in *Tgfb1* expression. No change was noted in death repressor gene *Bcl2.* But the significant increase in the expression of *Bcl2l1* may inhibit programmed cell death. Its overexpression is also a candidate that could contribute to pleiotropic pathways associated with hyperuricemia and chronical renal diseases [60].

### Gene Expression Changes via Transcription Factor Mechanisms Related to Kidney Injury Pathways

Epigenetic control of gene expression is significantly modulated through the regulation of transcription factors. The interaction between transcription factors and histone or DNA localizes the epigenetic regulatory machinery to regulate the expression of certain gene classes [61]. As alteration in expression was observed in many downstream pathways and kidney injury markers, we hypothesized that their changes may be related to the changed expression of nuclear receptor transcription factors known to be important for kidney homeostasis. We further examined the gene expression of five nonnuclear receptor transcription factors (Fig. 3B). A significant decrease in the expression of *Smarca2*/*Brm* after high-dosage treatment was observed. BRM is an SWItch/Sucrose Non-Fermentable (SWI/SNF) chromatin remodeling complex subunit and a crucial tumor suppressor in many cancers [62]. Histone deacetylase (HDAC) inhibitors were reported to restore BRM expression therefore inhibiting tumor growth. Although not significant, our study showed increased expression of *Atf3* after all treatment conditions. *Atf3*/*Lrg21* encodes cyclic AMP-dependent transcription factor ATF3, whose mRNA expression increased in response to mouse renal ischemia/reperfusion injury according to Kieran *et al*. [63]. ATF3 also recruits HDAC1 to ATF/NF-Κb sites in the promoters of inflammatory cytokines [64]. Nrf2/Nfe2l2 is thought to play a key role in the regulation of inflammatory responses against oxidative stress [65]. NFE2L2 is also a potential prognostic biomarker for kidney renal clear cell carcinoma [66]. Our data are supported by previous work by Stanifer *et al*. on PFAS exposure-related pathogenic pathways that upregulate *Nfe2l2* [67]. *Hes1* upregulation was found in acute kidney injury in an ischemia reperfusion model and correlated with the Notch signaling pathway [68]. Furthermore, the downregulation of the Hes1 signaling pathway was associated with decreased renal injury in rats [69].

As transcription factors, nuclear receptors are ligand regulated and can regulate cell homeostasis and metabolism. PPARs have been studied intensively due to their mechanistic effect of PFAS-induced toxicity. Our study showed significant *Ppara* upregulation, *Ppard* upregulation, and *Pparg* downregulation in expression after PFOS treatment at certain dosage levels (Fig. 3C). Ishibashi *et al*. found a dose dependency in PFOS-induced PPARα-mediated transcriptional activity in Baikal seals [70]. To the best of our knowledge, we are the first to report a positive correlation between *Ppard* expression and PFOS exposure in kidney. A recent study by Kobayashi *et al.* [71] in pregnant women also showed an interaction between maternal PFOS exposure and PPARD related to changes in maternal fatty acid levels. Previous studies support that transcription factors induce changes in the expression of other genes. For example, adipogenesis can be induced by PPARG [59], similar to the results observed in the PPAR pathway analysis in our study (Fig. 5C and D). Interestingly, decreased expression of *Pparg* was observed. Since the upregulation of PPARγ can induce mitochondrial stabilization to protect against oxidative stress [72], its downregulation could result in further oxidative damage in the kidney. Apoptosis occurred in cultured podocytes injured with puromycin aminonucleoside associated with decreased PPARγ mRNA expression [73]. Although not significant in post hoc test, the mRNA expression of *Nr1h2* did show a consistent increase in 5- and 10-mg/kg/day-treated mouse kidney. NR1H2/LXR is involved in the regulation of sterol and fatty acid metabolism. Deletion of both *LXRα* and *LXRβ* genes suppresses the entire class of enzymes involved in fatty acid and cholesterol metabolism mediated by insulin in the mouse [74]. LXRs are widely expressed in kidneys and responsible for lipid and cholesterol accumulation in glomeruli, as well as inflammation and oxidative stress in the kidney [75]. Our findings of PPAR expression alterations were further validated by GSEA pathway analysis.

### Potential Epigenetic Mechanisms

To study the mechanisms that lead to changes in the expression of transcription factors, which subsequently lead to altered metabolism pathways, as well as induction of chronic kidney diseases, we choose to examine the global DNA methylation changes and epigenetic regulators. Hypomethylation (42%) at 20 mg/kg/day dose was found to be statistically significant and aligns with our previous study on mouse liver exposed to PFOA [54]. Besides a potential causal relationship between altered global DNA methylation levels and toxicant exposure, we expect DNA methylation changes could potentially be used as a marker to measure the extent of damage to the cells upon exposure to the toxicant. Gene expression analysis was performed on six DNA methylation regulators, six histone methylation regulators, and eight histone acetylation regulators. No significant changes in expression were found in DNA methylation regulators and histone acetylation regulators. However, two histone methylation regulators were found to be upregulated significantly. After high-dosage treatment, *Kdm1a*/*Lsa1* expression increased significantly (Fig. 4C). Studies by Zhu *et al*. provided evidence that the overexpression of histone lysine-specific demethylase 1 (LSD1) was found in more than half of clear cell renal cell carcinomas [76]. Recent study also showed evidence that LSD1 could regulate renal cancer cell growth through epigenetic control of androgen receptor transcription factors [77]. *Kdm4c* expression increased significantly at low-dosage treatment. KDM4C was shown to promote tumor angiogenesis by transcriptionally activating the HIF1α/VEGFA signaling pathway [78]. Our data support the premise that histone methylation may play an important role in regulating transcription factors, including PPARs, contributing to alterations of the lipid and amino acid metabolism, as well as tumorigenesis and fibrosis pathways.

## Conclusions

Our study revealed both upregulation and downregulation of multiple kidney injury marker genes in PFOS-dosed mice. Along with GSEA, abundant information on altered pathways, including cellular processes such as fibrosis and tumorigenesis, molecular functions such as lipid and amino acid metabolism, and transcription regulation such as PPAR and TNF were also implicated. Concordance between PFOS-exposed kidney and a mutated mouse model that resulted in lethal kidney defects was found. Significant numbers of transcription factors including peroxisomal proliferator-activated receptors, *Ppara, Ppard*, and *Pparg*, were altered in gene expression. Our work suggests that PFOS-induced kidney injury and downstream phenotypic changes are due to epigenetic regulation mediated through transcription factors. Additionally, our study showed significant DNA hypomethylation and gene expression upregulation in two histone lysine-specific demethylases, *Kdm1a* and *Kdm4c*, indicating PFOS may inflict its damage to kidney cells through epigenetic alterations in DNA methylation and histones, providing cues to a new direction of inquiry. The opportunity to evaluate several other epigenetic regulators exist in future study. Further mechanistic studies on PFOS-induced epigenetic toxicity will shed light on its effects and role in metabolic disorders and cancer, particularly kidney cancer, where information is sparse and toxicity-specific biomarkers will be of significant benefit in health monitoring.

## Data Availability

Data available on request.
